# Attention-Guided Huber Loss for Head Pose Estimation Based on Improved Capsule Network

**DOI:** 10.3390/e25071024

**Published:** 2023-07-05

**Authors:** Runhao Zhong, Li He, Hongwei Wang, Liang Yuan, Kexin Li, Zhening Liu

**Affiliations:** 1 School of Mechanical Engineering, Xinjiang University, Urumqi 830046, China; 107552101383@stu.xju.edu.cn (R.Z.); wanghongwei@stu.xju.edu.cn (H.W.); yuanliang@mail.buct.edu.cn (L.Y.); li_kexin@stu.xju.edu.cn (K.L.); 107552103899@stu.xju.edu.cn (Z.L.); 2 School of Information Science and Technology, Beijing University of Chemical Technology, Beijing 100029, China

**Keywords:** head pose estimation, global attention block, self-attention routing, capsule network

## Abstract

Head pose estimation is an important technology for analyzing human behavior and has been widely researched and applied in areas such as human–computer interaction and fatigue detection. However, traditional head pose estimation networks suffer from the problem of easily losing spatial structure information, particularly in complex scenarios where occlusions and multiple object detections are common, resulting in low accuracy. To address the above issues, we propose a head pose estimation model based on the residual network and capsule network. Firstly, a deep residual network is used to extract features from three stages, capturing spatial structure information at different levels, and a global attention block is employed to enhance the spatial weight of feature extraction. To effectively avoid the loss of spatial structure information, the features are encoded and transmitted to the output using an improved capsule network, which is enhanced in its generalization ability through self-attention routing mechanisms. To enhance the robustness of the model, we optimize Huber loss, which is first used in head pose estimation. Finally, experiments are conducted on three popular public datasets, 300W-LP, AFLW2000, and BIWI. The results demonstrate that the proposed method achieves state-of-the-art results, particularly in scenarios with occlusions.

## 1. Introduction

The rapid development of artificial intelligence and the increasing prevalence of intelligent living have led to an increasing demand for robots to serve people in their daily lives. As an important component of service robot co-navigation, head pose estimation can provide information on human attention direction and intent, assisting robots in analyzing human behavior, and enabling them to possess the capability of social-aware navigation [1].

Head pose estimation refers to inferring the orientation of the head from a given image. It can be represented by a 3D vector that includes the pitch, roll, and yaw angles [2]. In recent years, extensive research on head pose estimation has driven the development of 3D reconstruction and interaction behavior analysis [3]. Head pose estimation has been widely used in many applications including virtual reality [4], fatigue driving detection [5], motion capture [6], and many other areas. In the driving system, it can determine the driver’s attention and consciousness based on position information [7]. Head pose is associated with visual attention, allowing semantic cues to be combined with dialogue and human interaction to facilitate non-verbal communication in special places, as well as being an important cue for predicting the direction of pedestrian movement. Therefore, it is used in many computer vision systems such as gaze discrimination [8], augmented reality [9], human–computer interaction [10], and surveillance security [11]. Chen [12] proposed a system for analyzing human behavior and human interaction in meetings and workplaces using head posture estimation.

Head pose estimation from an image requires learning a mapping of two-dimensional space and three-dimensional space. Traditional head pose estimation methods use face localization and image cropping to reduce the influence of image-independent backgrounds on the detection target, mainly by using face alignment to similarly match the target image to the image sample [13]. However, head pose estimation in complex environments is a challenging problem. To obtain an effective representation of facial features in partially occluded scenarios, Wu [14] extracted pyramid HoG features from non-occluded facial sub-regions to estimate head pose. Wang [15] improved the performance of the model by synthesizing head pose images and augmenting the sample images under different lighting and occlusion conditions. Xing [16] introduced occluded regions into facial appearance, recovered facial shape from partially occluded facial appearances, and modeled various types of partial facial occlusions. These methods are typically limited to individual head pose estimation and have low computational efficiency.

Head pose estimation utilizes a single RGB image since it can be regarded as a classification problem. Most of the methods proposed in the past five years for head pose estimation are based on machine learning and convolutional/deep neural networks [17]. Compared with other deep neural networks, the main contribution of Convolutional Neural Networks (CNN) is their ability to effectively replicate features from all positions in the input image’s spatial dimensions and use the learned features at other positions to achieve spatial reduction and local shared connectivity [18]. Nevertheless, CNNs often lack local equivariant features, resulting in weak generalization ability and loss of important object localization information, requiring additional parameters to construct deep networks. Hinton [19] proposed a novel form of cooperation between neurons using a new type of unit called capsules. In this unit, individual activations no longer represent the presence of specific features, but instead represent different properties of the same entity. Compared to traditional individual neurons, capsules consist of multiple neurons. On the convolution layer, the normal single-layer convolution is two-dimensional, while a single-layer capsule is three-dimensional. Each neuron within a capsule represents a specific attribute such as size, orientation, hue, texture, and other features. Consequently, capsules can capture more detailed information. As the head undergoes angular changes in the image, the neurons within the capsules also exhibit corresponding variations. Subsequently, a dynamic routing algorithm is employed to select and propagate capsules, thereby influencing the final output. Building on this idea, Sabor [20] proposed a capsule network that introduces capsules into the traditional CNN architecture. Each capsule represents a group of neurons that parameterize the instantiation parameters associated with different targets. Yang [21] proposed a head pose estimation method that employs capsule networks to filter candidate features and obtain representative features. However, the current capsule networks suffer from efficiency issues and inadequate representational capacity. They require many parameters, which inevitably obscure the intrinsic generalization ability that capsules should provide, leading to suboptimal accuracy in head pose estimation.

In this paper, we argue that head pose estimation methods suffer from low estimation accuracy and poor occlusion detection in complex environments. Therefore, we consider the limitations of the loss of spatial structure information and the inadequate generalization ability of capsule networks. To overcome these limitations, we propose a head pose estimation method based on an improved capsule network and attention-guided regression loss to enhance the accuracy of head pose estimation.

Specifically, the contributions of our work are as follows:

(1) We propose a detection model that combines CNN and capsule network for head pose estimation. The model includes feature extraction, feature mapping, and feature aggregation modules. A multi-level output structure backbone network is used to extract spatial structure and semantic information at different levels for improved estimation accuracy.
(2) In order to obtain more effective and representative features, we apply an improved feature extraction module to enhance the spatial weight of feature extraction, which can obtain more spatial information and significantly improve the feature extraction performance and network efficiency for capturing the main features.
(3) To address the problem of loss of spatial structure information, we utilize an improved capsule network with reduced number of capsules and parameters to enhance the network’s generalization ability. Moreover, we optimize the regression loss to improve the model’s robustness.
(4) To validate the effectiveness of the proposed method, we conduct tests and ablation experiments on the AFLW2000 and BIWI datasets. The results demonstrated that the performance of our method outperformed previous methods significantly.

## 2. Related Work

Head pose estimation, as a critical technology in computer vision, has attracted extensive attention and research. Various methods for head pose estimation have been proposed up to now based on different types of data including 2D color images, 3D images, and depth images. Methods based on 3D images and depth images require special equipment, such as depth cameras or stereo cameras, which are expensive and computationally complex, making real-time applications difficult [22]. Therefore, usually, only RGB images are utilized for estimating and analyzing head pose. In addition, previous methods using depth cameras are only accurate at close distances [23]. Depending on whether facial landmarks detection is required, head pose estimation methods are divided into landmark-based methods and landmark-freed methods.

### 2.1. Landmark-Based Methods

Facial landmark detection is used to establish the mapping relationship between 3D space and 2D images for estimating head pose [24]. Dlib [25] employed a collection of regression trees to locate facial landmarks with real-time prediction speed. 3DDFA [26] converted the head into a dense 3D model and uses a CNN to fit the 3D model to an RGB image. This method can effectively deal with occlusion problems. Nikolaidis [27] proposed a novel method for detecting facial landmarks using a combination of Adaptive Hough Transform (AHT) [28] and template matching techniques. The detected landmarks are then utilized to calculate the horizontal head pose, based on the deformation of an equilateral triangle formed by the landmarks of the two eyes and mouth. This method has shown promising results in accurately estimating the horizontal head pose in various scenarios. To improve the accuracy of head pose estimation, Narayan [29] proposed a universal geometric model for horizontal head pose estimation and validated its effectiveness on multiple standard datasets. The geometric-based approach has a simple process and low time complexity, requiring only a few facial features to obtain suitable head pose estimates. FAN [30], an advanced landmark detection method, obtains multiscale information by merging block features across layers and is robust to occlusion and head pose. KEPLER [31] uses a modified google net architecture to simultaneously predict facial key-points and poses. The coarse pose supervision methods are used to improve landmark detection.

The accurate estimation of head pose based on facial landmarks and model matching requires sufficient accuracy in both facial detection and feature point labeling. However, due to a high complexity, computational cost, and low efficiency of the model, it is difficult to accurately estimate head pose using classification training methods. In practical applications, the accuracy of facial feature point detection can be significantly reduced by various interfering factors such as lighting changes, complex backgrounds, head rotation, and occlusion [32], which can even make it impossible to detect facial feature points. Therefore, the model-based head pose estimation method is not entirely accurate.

### 2.2. Landmark-Freed Methods

The landmark-freed methods train on different samples of poses to obtain a vector description of the target pose sample, which represents a mapping between the pose and its features. By converting the head pose recognition problem into a classification or regression problem [17], it establishes a correspondence between the facial and head pose without relying on accurate facial feature point localization, enabling prediction of head pose with large deviations. Ruiz [33] proposed the Hopenet, which uses ResNet50 [34] as the backbone network to extract features and divides into three branches to jointly predict each angle. Each branch predicts the head pose angle by combining classification and regression objective functions. However, Hopenet performs coarse angle classification before aggregating regression, which introduces additional errors. To address this problem, Wang [35] proposed a hybrid coarse and fine classification framework introduced into this network, using more angle quantization units and other fine classifiers trained with auxiliary coarse units for better refinement of classification, which helps to reduce overfitting and improve the performance of prediction. Yang [21] proposed FSA-Net, which uses fine-grained structural mapping and scoring functions to filter important features and has a dual-stream multidimensional regression network based on the age classification algorithm SSR-Net [36]. By optimizing feature extraction and utilizing feature map aggregation of fine-grained structural mapping and scoring function to learn spatial relationships, it can estimate head pose without key-points information. Inspired by Hopenet, Zhou [37] proposed an end-to-end network model that changes the loss function and adapts to widely estimated training strategies to predict head pose angles across the entire range from a single image, which is the first method applicable to predicting head pose in the full range of head rotations. FDN [38] uses a feature decoupling model to explicitly identify the discriminative features for each angle by adaptively recalibrating the channel responses of each pose angle and suppressing less useful features. Zhu [39] proposed a hierarchical estimation method based on distinct network layers, gaining greater degrees of freedom in the angle estimation process. The LwPosr [40] uses a mixture of depth-separable convolution and Transformer [41] encoder layers with a dual-stream heterogeneous structure to extract features to provide fine-grained regression for predicting head pose. TriNet [42] considers orthogonal constraints on vectors to train the network, using three vectors to represent the head pose, and proposes Mean Absolute Error of Vectors to evaluate performance. There are also methods for estimating head pose based on multi-modal information. Gu [43] systematically analyzed the connection between Bayesian filtering and recurrent neural network (RNN) and use RNN to jointly estimate and track facial features in videos. Martin [44] proposed a method for head pose estimation on consumer depth cameras that combines head features and head model generation to build a detector that provides accurate results over a wide range of poses.

The deep learning-based models for head pose estimation employ an end-to-end recognition approach, which enables fast image processing, excellent high-dimensional feature extraction, strong generalization, and the extraction of the main features of head pose estimation tasks. However, this method faces the ambiguity of the Euler angle representation of head pose in a wide range of angles. Moreover, occlusion and multi-target detection significantly affect the accuracy of head pose estimation and result in the loss of target detection. 

## 3. Method

In this section, we first formulate head pose estimation problem (Section 3.1). Then, we give an overview of the proposed network (Section 3.2). In Section 3.3 and Section 3.4, we detail the feature extraction and feature mapping modules of the network. We illustrate the feature aggregation module and the use of SSR-Net to output the parameters predicted at each stage to obtain the final head pose estimation (Section 3.5). Finally, we optimize the regression loss function (Section 3.6).

### 3.1. Problem Formulation

Typically, head pose estimation can be represented as a regression problem-based image. We use a set of trained facial images $\left\{\left(x_{1},\;y_{1}\right),\left(x_{2},\;y_{2}\right),\ldots ,\left(x_{n},\;y_{n}\right)\right\}$ and the pose vector $y_{i}$ for each image $x_{i}$, where i is the number of images. Each head pose vector $y_{i}\;$can be subdivided into three angles: yaw, pitch, and roll. The goal of the regression task is to find a mapping function F and then use $\tilde{y}=F\left(x\right)$ to predict the head pose angle of the input image. We find F by minimizing the Mean Absolute Error (MAE) between the predicted attitude and the ground truth attitude. Also, we use MAE to assess the performance of all methods, calculating MAE for yaw, pitch and roll separately and then averaging them for overall evaluation.

(1) $$J\left(x\right)=\frac{1}{N}\sum_{n=1}^{N}\|{\tilde{y}}_{n}-y_{n}\|\;,$$

where ${\tilde{y}}_{n}=F\left(x_{n}\right)$ is the predicted pose for the trained image $x_{n}$. $J\left(x\right)$ is a function of the reduced MAE.

### 3.2. Overview of Proposed Network

**Figure 1:**
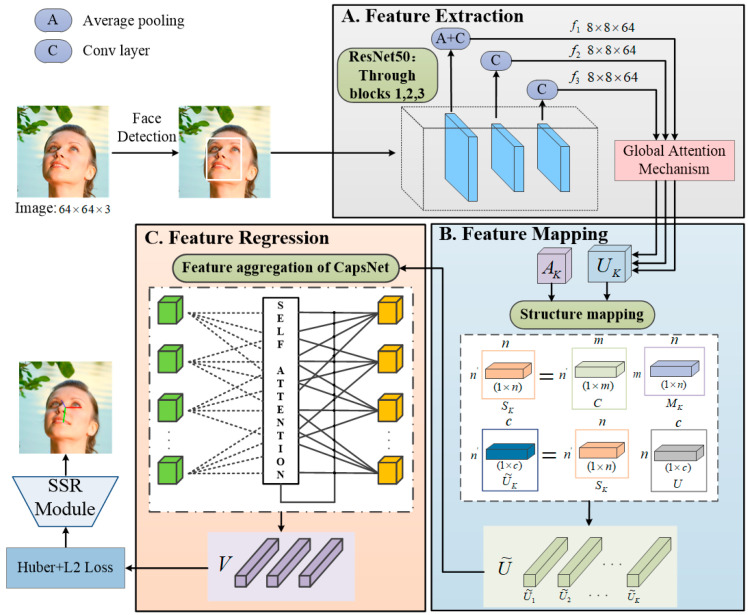
Overview of the proposed network.

The proposed network model is illustrated in Figure 1, which combines deep residual networks and improved capsule networks. It consists of three main components: the feature extraction module, feature mapping module, and feature aggregation module. For the task of feature extraction, both Hopenet [33] and TriNet [42] employ ResNet50 as the backbone network and have demonstrated good performance in various experiments. We employ the same backbone network to minimize unrelated variables and ensure a fair comparison.

Firstly, we employ multitask cascaded convolution neural network (MTCNN) [45] to detect the face region in the input image. Then, we feed the image into a feature extraction module, which adopts the Resnet50 with a multi-level output structure to extract feature maps from three stages and obtain spatial and semantic information at different levels. A global attention block (GAB) [46] is introduced in each stage to enhance the feature weight and extraction ability of key information and obtain more spatial information. The feature mapping module can obtain a more representative feature set. Next, we apply the improved capsule network to the feature aggregation module to obtain the final feature set. We optimize the regression loss function to enhance the robustness of the model. Finally, we use SSR-Net to output the predicted parameters from each stage and obtain the final head pose estimation result. 

### 3.3. Feature Extraction

#### 3.3.1. Face Detection

**Figure 2:**
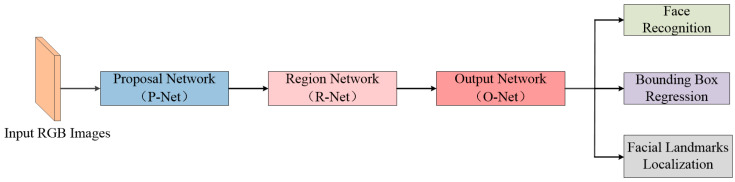
Architecture of MTCNN for face detection.

Detecting faces and face alignment from images is challenging in varying unconstrained environments. Different lighting conditions, visual variations of faces and extreme head pose variations are the main challenges in correctly detecting faces from images [47]. Therefore, we use MTCNN as a face detector to detect faces and obtain their bounding box coordinates. It provides a real-time solution for detecting human heads across various scales and angles, even in the presence of complex and cluttered backgrounds. This capability is crucial for practical applications, where the quick and accurate detection of human heads is required. Figure 2 shows the architecture of the MTCNN. It is a network structure consisting of three layers of cascaded CNNs (P-Net, R-Net and O-Net). It utilizes bounding box regression and non-maximum suppression candidate filters to calibrate each layer of the network. The refined layer is more precise than the former, and the network parameters are trained to perform multiple tasks, resulting in a straightforward-to-advanced face detection process.

#### 3.3.2. Global Attention Block

**Figure 3:**
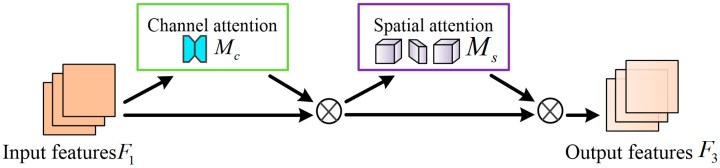
Architecture of the GAB.

In deep convolution neural networks, attention mechanisms can refine feature maps to achieve better performance. To extract more effective features from the input face image, we design to introduce an efficient GAB that reduces information loss and amplifies cross-spatial-channel interactions across all three dimensions of importance. This module can improve the extraction efficiency of key features and the performance of deep neural networks. The structure diagram of GAM is shown in Figure 3. GAB captures global cross-space channel interactions, aiming to ensure the effectiveness and interactivity of feature information. It adopts the sequential channel-space attention mechanism from the convolution block attention module [48] and redesigns its sub-modules. The specific process can be represented by Equations (2) and (3). Given the input map $F_{1}\in R^{C\times H\times W}$, the intermediate state $F_{2}$ and the output $F_{3}$ are defined as:

(2) $$F_{2}=M_{c}\left(F_{1}\right)⊗F_{1},$$

(3) $$F_{3}=M_{s}\left(F_{2}\right)⊗F_{2},$$

where $M_{c}$ and $M_{s}$ are the channel and spatial attention maps; ⊗ denotes element-based multiplication.

**Figure 4:**
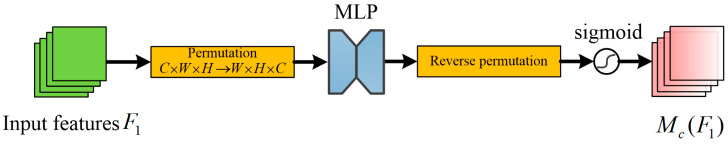
Architecture of the channel attention sub-module.

The channel-attention sub-module uses 3D alignment to retain information, and then it uses a multi-layer perceptron to amplify the channel-space dependencies across dimensions. Finally, the original alignment is recovered, and a set of channel weights is generated after a sigmoid activation function to represent the weights of the feature mapping between channels. An enhanced feature map can be obtained by multiplying the weights with the input feature map. The channel attention sub-module is illustrated in Figure 4.

**Figure 5:**
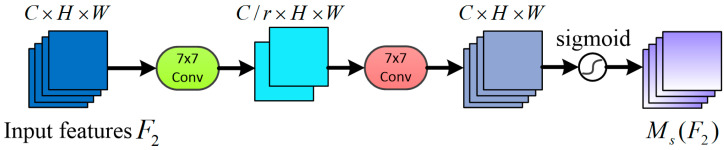
Architecture of the spatial attention sub-module.

In the spatial attention sub-module, two convolution layers are used for spatial information fusion and the same reduction ratio as in the channel attention sub-module to focus spatial information. Also, pooling is removed to further preserve feature mapping. The spatial attention sub-module is illustrated in Figure 5.

### 3.4. Feature Mapping

**Figure 6:**
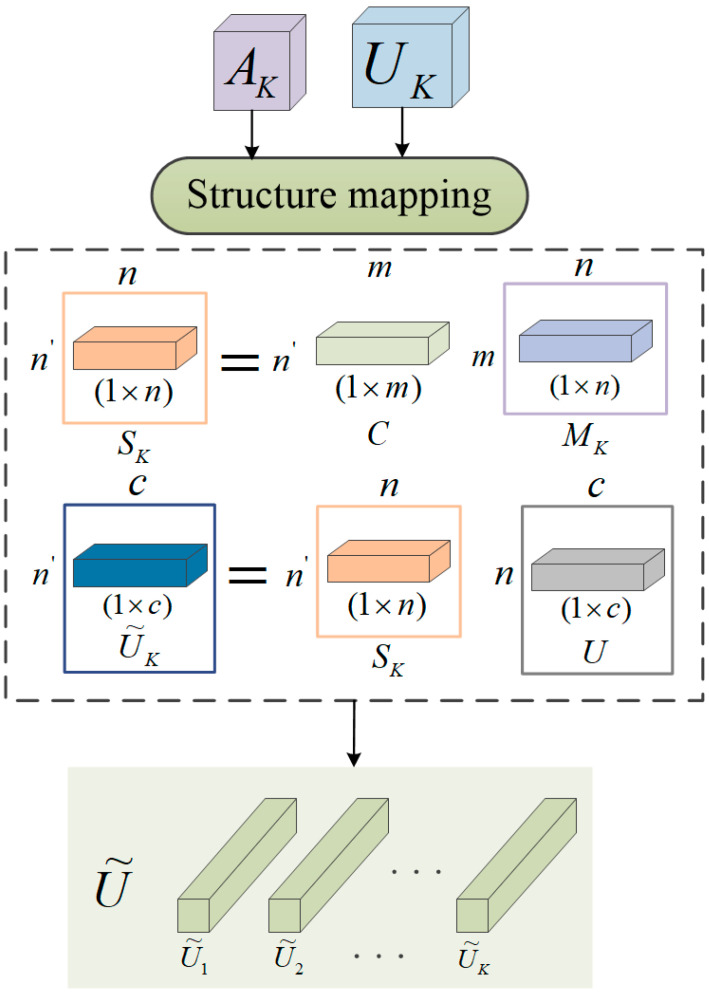
Architecture of the feature mapping model.

After the above feature extraction module, we obtain the feature maps $U_{k}$, whose dimension is w×h×c. Figure 6 illustrates the structure of the feature mapping module. To obtain smaller and representative feature maps, a scoring function $\phi \left(u\right)$ is used to measure their importance. Similar to [21], we force the fine-grained mapping module. The function $\phi \left(u\right)\;$includes 1×1 convolution, variance, and uniform to facilitate spatial grouping. For each feature map $U_{k}$, we obtain an attention map $A_{k}$ by Equation (4).

(4) $$A_{k}=\phi \left(U_{k}\left(i,j\right)\right).$$

The next step is to perform fine-grained structure mapping to obtain more representative features $\tilde{U}$. Figure 6 illustrates the process. All feature maps are first flattened into a 2D matrix U, where $U\in R^{n\times c}$ and n=w×h×k. The matrix U contains all the pixel-level features in all phase feature maps. For the k-th stage, we design a mapping $S_{k}$ to extract $n^{\prime }$ representative features ${\tilde{U}}_{k}$ by Equation (5).

(5) $${\tilde{U}}_{k}=S_{k}\times U,$$

where $S_{k}\in R^{n^{\prime }\times n}$ and ${\tilde{U}}_{k}\in R^{n^{\prime }\times c}$. In other words, we obtain a representative feature by a linear combination of pixel-level features. Mapping $S_{k}$ is a linear transformation that performs linear dimensionality reduction by a weighted average of all pixel-level features. The mapping $S_{k}$ is the multiplication of C and $M_{k}$. The maps $M_{k}$ and C are formed as follows:

(6) $$M_{k}=\sigma \left(f_{M}\left(A_{k}\right)\right),$$

(7) $$C=\sigma \left(f_{C}\left(A\right)\right),$$

where $C\in R^{n^{\prime }\times m}$ and $M_{k}\in R^{m\times n}$ is the sigmoid function; $f_{M}$ and $f_{C}$ are two different functions defined by fully connected layers. $A=\left[A_{1},A_{2},\ldots ,A_{k}\right]$ is the concatenation of all attentive maps. Finally, all features ${\tilde{U}}_{k}$ are connected to form a final set of representative features $\tilde{U}=\left[{\tilde{U}}_{1},{\tilde{U}}_{2},\ldots ,{\tilde{U}}_{k}\right]$, where $\tilde{U}\in R^{\left(n^{\prime }\times k\right)\times c}$.

### 3.5. Feature Aggregation

Regarding the aggregation module, we first consider CNNs because their convolutional structures can effectively capture existing features. However, CNNs rely on large amounts of data and layers with feature mappings to complete learning and updates, which may not be very efficient. When capturing relationships between feature attributes, CNNs may cause feature detectors to lose the precise target information from input images. Capsule networks provide a good solution, as they can extend current convolutional networks to efficiently encode all affine transformations of features and have better generalization capabilities.

To overcome the limitations of the CNN method, we adopt the capsule network for feature aggregation. However, the capsule network requires training a large number of parameters, leading to low utilization efficiency and insufficient inherent generalization ability in expressing feature transformations. To address this problem, we use the linear combination and self-attention routing mechanism (SARM) [18]. Utilizing the method that can acquire more comprehensive features can enhance the network’s capability in facial feature extraction, thereby decreasing the effect of absent facial feature information on the forecasting outcomes. At the same time, it also effectively reduces the number of capsules, enhances the generalization ability of the capsule network and improves the network’s recognition performance for head posture.

**Figure 7:**
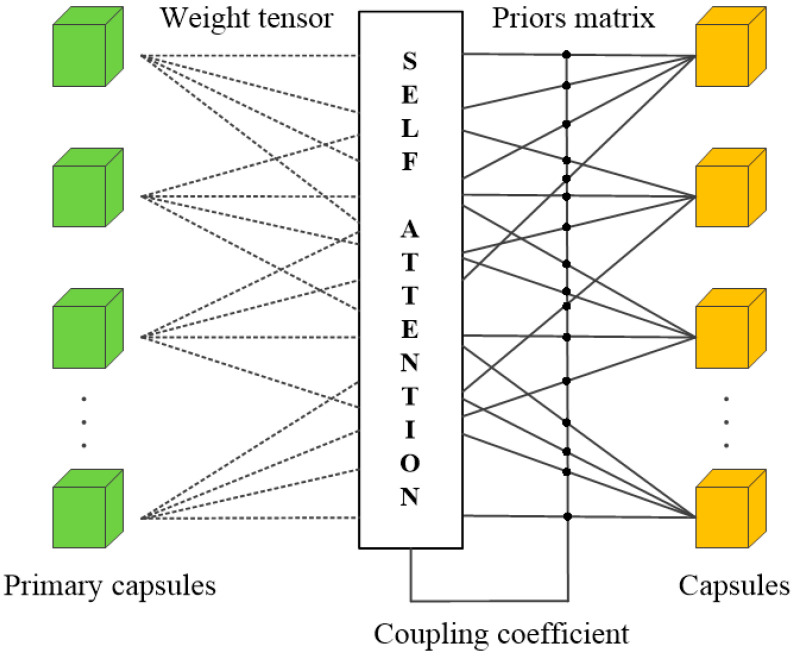
Architecture of the SARM.

As shown in Figure 7, the mechanism is similar to a fully connected network with additional branches introduced by the self-attention algorithm. In fact, the upper-level capsules receive the total input as a weighted sum of the “predicted vectors” from the lower-level capsules. The weight matrix is obtained by matrix multiplication for each capsule. The tensor that contains all the weight matrices is then embedded into the affine transformations between adjacent capsules, allowing the lower-level capsules to predict the properties of all upper-level capsules. This tensor mainly consists of the log prior matrix and the coupling coefficient matrix. The log prior matrix includes all the weights that are learned discriminatively with other weights, which helps to establish more closely related capsules. The coupling coefficient matrix is a matrix that contains all the coupling coefficients generated by the self-attention algorithm. The self-attention routing dynamically assigns the detected shape to the represented entity. Therefore, the lower-level capsules can efficiently aggregate features by predicting the upper-level capsules through this mechanism, reducing the number of capsules and trainable parameters, and obtaining better results.

### 3.6. Optimization of Loss Functions

For the loss function of the regression model, TriNet [42] employs the Mean Square Error (MSE) loss function for both regression and orthogonal losses. The gradient of MSE decreases as the error decreases, which is beneficial to the convergence of the model. However, the MSE loss function is susceptible to the influence of outliers, causing the regression line to shift towards the outlier data points. FSA-Net [21] uses the MAE loss function, which has a stable gradient and does not cause gradient explosion. Moreover, it is less affected by outliers and has greater inclusivity, which allows the fitted line to better characterize the distribution of normal data. In most cases, the gradient of MAE remains constant, which implies that even for small losses, the gradient still holds a significant value. This characteristic could impede function convergence and the learning of the model.

To address this problem, we propose a novel regression loss function $L_{hul}$ that incorporates Huber loss [49] and L2 regularization loss. To the best of our knowledge, this is the first application of Huber loss to head pose estimation. Huber loss is a loss function that combines MSE and MAE, with a hyperparameter α that determines the weight between the two components. When $\left|g-f\left(t\right)\right|\leq \alpha$, Huber loss becomes MSE. When $\left|g-f\left(t\right)\right|>\alpha$, Huber loss approximates to MAE. Huber loss, therefore, offers the advantages of both MSE and MAE, reducing the problem of sensitivity to outliers. Also, we use L2 regularization loss to ensure the generalization of the model, as described in Equation (8).

(8) $$\;L_{hul}=\left\{\begin{array}{c} \frac{1}{2}{\left(g-f\left(t\right)\right)}^{2}+\lambda {\left\|w\right\|}_{2}{}^{2},\left|g-f\left(t\right)\right|\leq \alpha \\ \alpha \left|g-f\left(t\right)\right|-\frac{1}{2}\alpha^{2}+\lambda {\left\|w\right\|}_{2}{}^{2},\left|g-f\left(t\right)\right|>\alpha \end{array}\right.\;,$$

where g is the sample true pose, $f\left(t\right)$ is the prediction pose, λ is the regularization factor, and w is the regularization parameter.

## 4. Experiments

In this section, we provide a detailed account of the experimental procedure and results. The first part describes the experimental setup, while the second part introduces the dataset and evaluation metrics used in the experiments. The third part presents experimental results to test the effectiveness of the proposed network. Finally, we compare the experimental results with those of typical head pose estimation algorithms to further validate the performance of our method.

### 4.1. Experimental Implementation

The experiments are performed on a computer with an Intel Xeon Gold 5118 CPU and Nvidia RTX 5000 GPU. We use Keras with TensorFlow 1.10.0 backend to implement the proposed network. To make a fair comparison, we apply random cropping and random scaling to training images by following the data augmentation strategies in training from FSA-Net [21] and TriNet [42]. We apply Adam [50] as the optimizer for training with the initial learning rate 0.001. We use 100 epochs to train the network and the learning rate is reduced by a factor of 0.1 every 30 epochs. To improve the processing of blurred and enlarged images, random cropping and random scaling are used on the training images to enhance the training data. 

### 4.2. Datasets and Experimental Protocols

**Figure 8:**
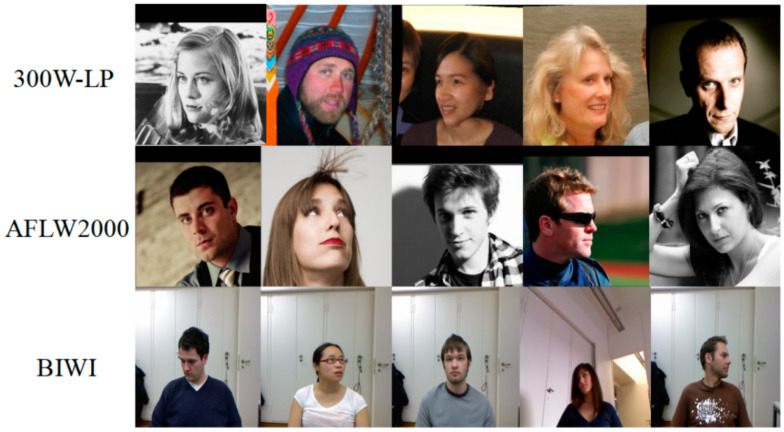
Sample images from the three datasets.

In Figure 8, the experiments used three datasets for head pose estimation: 300W-LP [26], AFLW2000 [51], and BIWI [52].

**300W-LP:** The 300W-LP dataset is a combined dataset containing eight small datasets. It has a total of 122,450 images and contains samples of large-angle head poses, and the images are labeled with the Euler angles rotated during the image processing. This dataset is currently the largest publicly available dataset in the field of head pose estimation and has been chosen for the training of head pose estimation models proposed in recent years.

**AFLW2000:** The AFLW2000 dataset consists of 2000 images selected from the AFLW dataset, with most of its sample being portraits of people in natural scenes. The images in the AFLW2000 dataset include variations in the pose of people in different scenes and brightness, covering people of different ages and ethnicities. It has pretty accurate face pose annotation providing ground-truth 3D faces and the corresponding 68 landmarks. Therefore, we test the model using the AFLW2000 dataset to verify the generalization capability of the model.

**BIWI:** The BIWI dataset is a publicly available dataset commonly used in the field of head pose estimation, with approximately 15,000 images. The dataset contains 24 videos of head poses from 20 test subjects (6 females and 14 males). In addition to RGB frames, the dataset also provides the depth image for each frame. Ground-truth is provided in the form of the 3D location of the head and its rotations.

For comparison with state-of-the-art methods, we follow the same training and testing setup as mentioned in Hopenet [33], FSA-Net [21], and TriNet [42]. For training and testing on these datasets, we implement our experiments in two common protocols:

(1) In our first protocol, we follow the convention by using the 300W-LP dataset for training and AFLW2000 and BIWI datasets for testing. When evaluating on the BIWI dataset, we do not use tracking and only consider using MTCNN [45] face detection samples whose rotation angles are in the range of [−99°, +99°] to keep consistent with the strategies used by Hopenet [33], FSA-Net [21] and TriNet [42]. Also, we compare several state-of-the-art landmark-based pose estimation methods using this protocol.
(2) For the second protocol, we follow the convention by FSA-Net [21] and randomly split the BIWI dataset in a ratio of 7:3 for training and testing, respectively. The train set is not crossed with the test set. MTCNN [45] uses experience tracking technology to detect faces in the BIWI datasets, avoiding the failure of face detection. This protocol is used by several pose estimation methods such as RGB, depth, and time, whereas our method uses only a single RGB frame.

In all the experiments above, we evaluate the performance of all methods using MAE. For each method, MAE for yaw, pitch, and roll is separately calculated and then taking the average for overall evaluation.

### 4.3. Experiment Results

We explore the performance variation of the model using different loss functions, namely MAE, Huber loss and $L_{hul}$ loss. Table 1 shows the comparative results of the three different loss functions for protocol 1. 

We test each of the three loss functions by incorporating them into the improved model, where “×” indicates that the improved model is not used and “√” indicates that the improved model is used. The result shows that the improved model can improve performance by incorporating different loss functions. We observe that there is little difference in performance between both using MAE and Huber loss. We analyze that the use of Huber loss leads to overfitting of the model. We propose that $L_{hul}$ can be a good solution to the problem and greatly improve the performance of the model.

#### 4.3.1. Results with Protocol 1

In protocol 1, the features of the training and test datasets are completely different, with the training dataset being synthetic and the test dataset being real. The landmark-free approach can better accommodate the domain differences between training and testing. As a result, the landmark-free approach performs better than the landmark-based approach on both the AFLW2000 and BIWI datasets.

Table 2 and Table 3 compare the proposed method with the state-of-the-art methods on the AFLM2000 and BIWI datasets, respectively. As shown in Table 2, the proposed method realizes the best performance and attains the minimum error on yaw when tested on the AFLW2000 dataset. Pitch and roll angle errors are somewhat higher than TriNet, but an average deviation angle error reduction of 0.07 compared to TriNet. Furthermore, in Table 3, the experimental results on the BIWI dataset are shown. The proposed method reaches minimum error on a roll, and other indicators are also in the upper middle position with an average deviation angle error of 3.93.

#### 4.3.2. Results with Protocol 2

Table 4 compares the performance of other state-of-the-art methods on the BIWI dataset, where 70% and 30% of the data are randomly splatted for training and testing, without crossover. However, TriNet [42] applies a 3-fold cross validation on BIWI dataset in this protocol. It split the dataset into three groups and ensures that the images of one person should appear in the same group. For a fair comparison, we return the open-sourced models and measure the results of MAE under the same experimental protocol. The BIWI dataset contains multiple modes of information, and in addition to RGB information, depth or temporal information can be used to improve performance. The finding of methods based on multi-modal information is derived from FSA-Net [21]. The results show that our model does not perform as well as methods using multiple modalities on pitch and roll, but it only uses a single RGB frame and outperforms all other methods in its peer group. In addition, our method achieves the best yaw angle estimation and overall performance, even outperforming methods that utilize multiple modalities. 

#### 4.3.3. Results with Model Size and Computation Time

To fully understand the performance of the proposed network, we compare the proposed method with other state-of-the-art methods in terms of model size, parameters, and computation time. For a fair comparison, all networks are tested on the same target platform. Table 5 shows that our method achieves a computation time of about 12 fps, which outperforms other state-of-the-art methods. Our method is larger than FSA-Net in terms of model size and parameters but performs better in head pose estimation. Moreover, compared to Hopenet [33] and TriNet [42], our method applies the same backbone network but has a smaller model size and parameters.

### 4.4. Visualization

**Figure 9:**
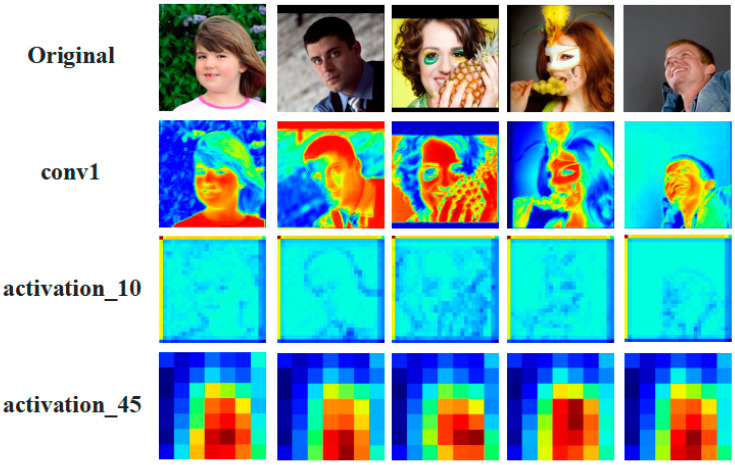
The feature map visualizations of head pose with occlusion and significant angle deflection on the AFLW2000 dataset.

Figure 9 presents the visualizations of feature maps extracted by the feature extraction module at different stages. First, coarse features are extracted from the input image. Then, through adjusting channel sizes and performing consecutive convolution and pooling operations, deeper-level features are extracted from the outputs of the previous stage. Finally, redundant information is removed from the features, while preserving the relevant information to enhance the efficiency of the network. The results demonstrate that the proposed method effectively captures representative features for occluded and angle-deflected images.

**Figure 10:**
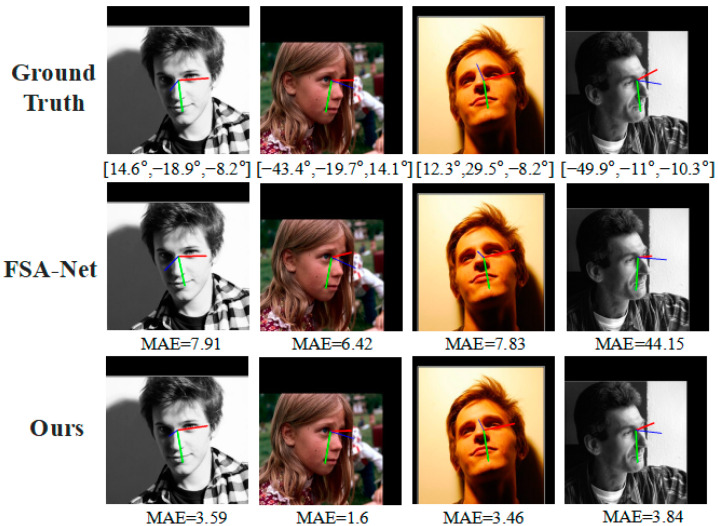
Estimation of head pose with significant angle deflection on the AFLW2000 dataset.

To further validate the effectiveness of our proposed method, we present a visualization comparison between different methods. Figure 10 shows the head pose estimation results of the proposed method and FSA-Net on images with significant angle deviations. Three different colored lines are used to represent pitch, yaw, and roll directions, respectively, making the head pose estimation results visualizable. Specifically, the blue line indicates the direction of the face, the green line indicates the direction of the bottom, and the red line indicates the direction of the side.

**Figure 11:**
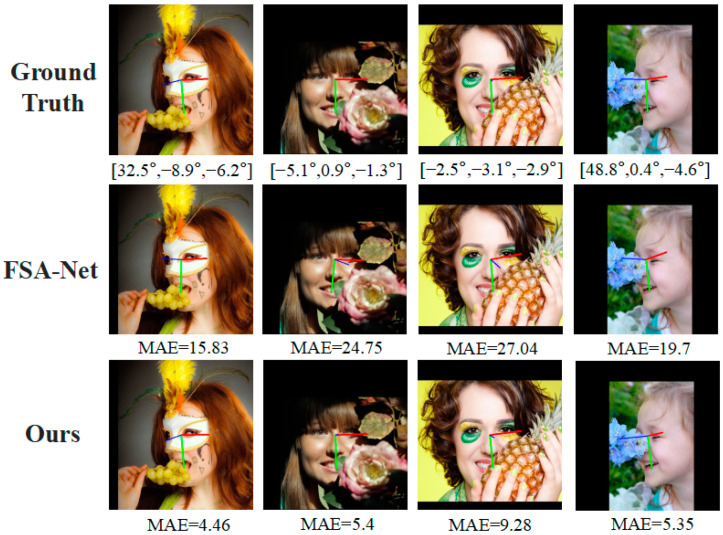
Estimation of head pose with occlusion on the AFLW2000 dataset.

In this study, we propose a method for head pose estimation that shares a similar feature mapping module with FSA-Net. To demonstrate the effectiveness of our proposed method, we select a subset of images with significant occlusions from the AFLW2000 dataset. Figure 11 shows some challenging examples. The visualization analysis results indicate that our proposed method is closer to the ground truth labels in cases of significant angle deviation and occlusions compared to FSA-Net.

**Figure 12:**
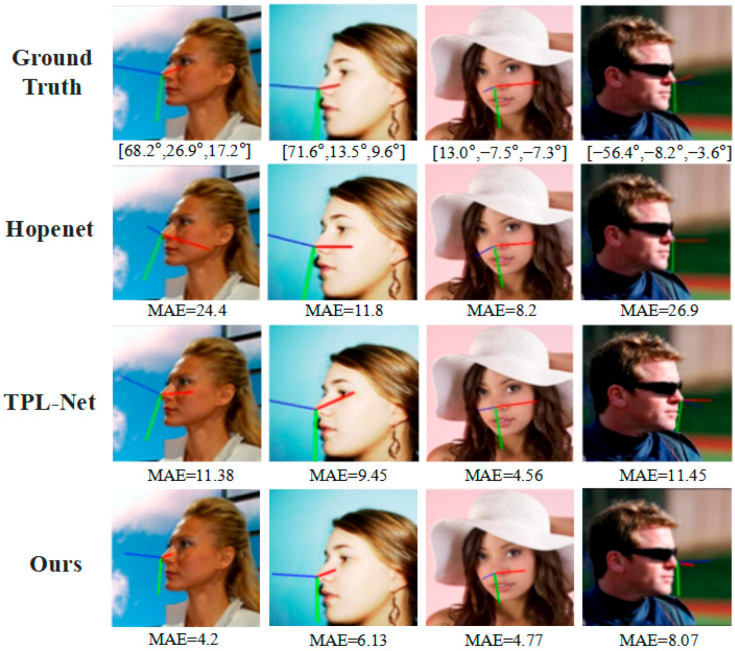
Various methods for estimating head pose sample images on the AFLW2000 dataset. The input images and results of other methods from (Zhu 2022 [39]).

In addition, to strengthen the persuasive power of our experiments, we refer to the visualization results of Zhu [39]. Based on their work, we compare our proposed method with other methods. The results in Figure 12 show that our method achieves the best performance.

### 4.5. Ablation Study

In this section, we conducted ablation experiments to investigate the impact of different modules (feature extraction module, feature aggregation module, and regression loss function) on the performance of our proposed model. The proposed method was trained on the 300W-LP dataset and tested on the AFLW2000 and BIWI datasets for the three modules. We then used 70% of the BIWI dataset as a training set and 30% as a test set. The experimental results for the different datasets are shown in Table 6 and Table 7, respectively.

Table 6 shows the results of the ablation experiments for the proposed method on the AFLW2000 and BIWI datasets. The baseline model achieves an MAE of 5.17 on the AFLW2000 dataset and an MAE of 4.24 on the BIWI dataset when the proposed modules are not used. From the results of adding each of the three modules separately, it can be seen that each module improves the performance of the model, with SARM having the best performance on the AFLW2000 dataset. We analyzed that this mechanism improves the performance and generalization ability of the capsule network by reducing the number of capsules and trainable parameters, effectively avoiding the problem of spatial structural information loss. When all modules are combined, the proposed model achieves an MAE of 4.60 on the AFLW2000 dataset and an MAE of 3.93 on the BIWI dataset. This indicates that all three proposed modules are effective.

**Figure 13:**
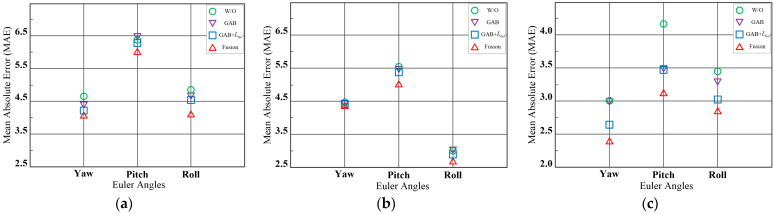
Comparison of the MAE of various components at various angles under protocol 1 and protocol 2. We divide the components of the proposed method into three parts, GAB, GAB fusion $L_{hul}\;$loss, and all modules fused. “w/o” denotes without the proposed method. (**a**) AFLW2000 (protocol 1); (**b**) BIWI (protocol 1); (**c**) BIWI (protocol 2).

Table 7 presents the ablation study results of our proposed model based on RGB information on the BIWI dataset, which includes depth information of face images and head poses with different angles. Compared to the baseline model, our proposed method reduces the MAE by 0.95, indicating its suitability for head poses with different angle ranges. Figure 13 provides a detailed comparison of yaw, pitch, and roll angles under several settings of the ablation study. The best experimental results are obtained when all three modules are combined, demonstrating that these modules together enhance the accuracy of head pose estimation, significantly improving the performance of head pose estimation algorithms, and thus validating the effectiveness of our proposed method.

## 5. Conclusions

To avoid loss of spatial information and improve the estimation accuracy of head pose under occlusion, in this paper, we propose a head pose estimation model based on a fusion of a convolutional neural network and capsule network. We adopt a feature extraction network with a multi-level output structure and introduce GAB to enhance the spatial weight of feature extraction, which can obtain more spatial information. The capsule network can retain and transmit spatial information. We improve the capsule network with SARM to significantly reduce the number of capsules and trainable parameters, effectively improving the model’s performance and solving the problem of loss of spatial structural information. In addition, to enhance the robustness of the model, we have utilized Huber loss for head pose estimation for the first time, which has better performance compared to methods based on multiple loss functions. The collaborative fusion of convolutional neural networks and capsule networks facilitates the extraction and preservation of both spatial and semantic features, resulting in better detection of head pose.

This study conducts experiments on AFLW2000 and BIWI datasets, comparing the proposed method with previous methods. The results demonstrate that the proposed model exhibits more advanced performance in cases of significant angle deviations and occlusions. In future work, we will primarily focus on real-time head pose detection for analysis in social interactions. To achieve this, we will investigate lightweight network architectures to further improve computational speed and optimize the model parameters. Additionally, we intend to incorporate multimodal information, such as depth and temporal cues, for head pose estimation, with the aim of enhancing the performance of the network.

## Figures and Tables

**Table 1:** Performance comparison between different loss functions on the AFLW2000 and BIWI datasets. All are trained on the 300W-LP dataset. The bolded data is the most effective.

Loss	Improved	AFLW2000				BIWI			
		Yaw	Pitch	Roll	MAE	Yaw	Pitch	Roll	MAE
MAE	×	4.49	6.22	4.64	5.12	4.39	5.36	2.76	4.17
MAE	√	4.00	6.17	4.27	4.81	4.36	4.89	2.64	4.05
Huber loss	×	4.12	6.37	4.58	5.02	4.51	5.16	2.71	4.13
Huber loss	√	3.94	6.13	4.27	4.78	4.42	4.90	2.59	3.97
$L_{hul}$ loss	×	4.03	6.21	4.38	4.87	4.34	5.02	2.70	4.02
$L_{hul}$ loss	√	**3.91**	**5.78**	**4.11**	**4.60**	**4.25**	**4.96**	**2.57**	**3.93**

**Table 2:** Comparisons with the state-of-the-art methods on the AFLW2000 dataset. All are trained on the 300W-LP dataset. The bolded data is the most effective.

Method	Yaw	Pitch	Roll	MAE
Dlib [25]	23.10	13.60	10.50	15.80
FAN [30]	6.36	12.3	8.71	9.12
3DDFA [26]	5.40	8.53	8.25	7.39
Hopenet [33]	6.47	6.56	5.44	6.16
SSR-Net-MD [36]	5.14	7.09	5.89	6.01
FSA-Net [21]	4.50	6.08	4.64	5.07
WHENet [37]	5.11	6.24	4.92	5.42
TriNet [42]	4.20	**5.77**	**4.04**	4.67
LwPosr [40]	4.80	6.38	4.88	5.35
Ours	**3.91**	5.78	4.11	**4.60**

**Table 3:** Comparisons with the state-of-the-art methods on the BIWI dataset. All are trained on the 300W-LP dataset. The bolded data is the most effective.

Method	Yaw	Pitch	Roll	MAE
Dlib [25]	16.80	13.80	6.19	12.2
FAN [30]	8.53	7.48	7.63	7.89
3DDFA [26]	36.20	12.30	8.78	19.10
Hopenet [33]	4.81	6.61	3.27	4.90
KEPLER [31]	8.80	17.3	16.2	13.9
SSR-Net-MD [36]	4.49	6.31	3.61	4.65
FSA-Net [21]	4.27	4.96	2.76	4.00
TriNet [42]	**3.05**	**4.76**	4.11	3.97
LwPosr [40]	4.11	4.87	3.19	4.05
Ours	4.25	4.96	**2.57**	**3.93**

**Table 4:** Comparisons with the state-of-the-art methods on the BIWI dataset. 70% of videos are trained and 30% for testing. The bolded data is the most effective.

Method	Input	Yaw	Pitch	Roll	MAE
SSR-Net-MD [36]	RGB	4.24	4.35	4.19	4.26
FSA-Net [21]	RGB	2.89	4.29	3.60	3.60
TriNet [42]	RGB	3.18	3.57	2.85	3.20
VGG16+RNN [43]	RGB + Time	3.14	3.48	**2.60**	3.07
Martin [44]	RGB + Depth	3.60	**2.50**	**2.60**	2.90
Ours	RGB	**2.37**	3.14	2.83	**2.78**

**Table 5:** Comparisons with the state-of-the-art methods in terms of model size, parameters, and FPS. The bolded data is the most effective.

Method	Image Size	Model Size (MB)	Parameters (M)	FPS
Hopenet [33]	224×224	95.9	23.92	9
FSA-Net [21]	64×64	**5.1**	**1.17**	6
TriNet [42]	64×64	27.96	1.95	10
Ours	64×64	21.3	1.68	**12**

**Table 6:** Ablation study for different feature extraction modules (with/without GAB) and capsule network (with/without SARM) and loss function (with/without $L_{hul}$ loss). All are trained on the 300W-LP dataset. The bolded data is the most effective.

GAB	SARM	$L_{hul}\;\mathrm{Loss}$	AFLW2000				BIWI			
			Yaw	Pitch	Roll	MAE	Yaw	Pitch	Roll	MAE
×	×	×	4.55	6.24	4.72	5.17	4.37	5.46	2.89	4.24
√	×	×	4.31	6.32	4.59	5.07	4.33	5.41	2.94	4.20
×	√	×	4.43	6.15	4.45	5.01	4.35	5.24	2.80	4.13
×	×	√	4.47	6.28	4.52	5.09	4.40	5.39	2.69	4.16
√	√	×	4.05	5.87	4.33	4.75	4.32	5.08	2.60	4.00
√	×	√	4.14	6.11	4.42	4.89	4.30	5.25	2.72	4.09
×	√	√	4.20	6.06	4.23	4.83	4.36	5.21	2.61	4.06
√	√	√	3.91	5.78	4.11	**4.60**	4.25	4.96	2.57	**3.93**

**Table 7:** Ablation study for different feature extraction modules (with/without GAB) and capsule network (with/without SARM) and loss function (with/without $L_{hul}$ loss). All are trained on the BIWI dataset (70%). The bolded data is the most effective.

GAB	SARM	$L_{hul}\;\mathrm{Loss}$	BIWI			
			Yaw	Pitch	Roll	MAE
×	×	×	2.96	4.43	3.80	3.73
√	×	×	2.91	3.45	3.21	3.19
×	√	×	2.56	3.48	3.19	3.07
×	×	√	2.73	3.53	3.07	3.11
√	√	×	2.45	3.25	2.90	2.87
√	×	√	2.60	3.41	2.91	2.97
×	√	√	2.39	3.27	2.87	2.84
√	√	√	2.37	3.14	2.83	**2.78**

## Data Availability

We use three publicly available datasets, 300W-LP, AFLW2000, and BIWI in our experiments. Their links are as follows: 300WLP: http://www.cbsr.ia.ac.cn/users/xiang yuzhu/projects/3DDFA/main.htm; AFLW2000: http://www.cbsr.ia.ac.cn/users/xiangyuzhu/prjects /3DDFA/main.htm; BIWI: https://data.vision.ee.ethz.ch/cvl/gfanelli/head_pose/head_forest.html (all of the above datasets accessed on 23 April 2023).
